# Association between gut microbiota and endometriosis: a two-sample Mendelian randomization study

**DOI:** 10.3389/fmicb.2023.1188458

**Published:** 2023-09-27

**Authors:** Xuan Ji, Qi Yang, Xiu-Lin Zhu, Li Xu, Jie-Ying Guo, Yan Rong, Yun-Lang Cai

**Affiliations:** ^1^Medical School of Southeast University, Nanjing, Jiangsu Province, China; ^2^Department of Obstetrics and Gynecology, Shanghai General Hospital, Shanghai, China; ^3^Department of Obstetrics and Gynecology, Zhongda Hospital, Medical School of Southeast University, Nanjing, Jiangsu Province, China

**Keywords:** endometriosis, gut microbiota, Mendelian randomization analysis, genome-wide association study, instrumental variables

## Abstract

**Background:**

Recent studies have shown that an imbalance in gut microbiota (GM) may not always be associated with endometriosis (EMS). To investigate this further, we conducted a two-sample Mendelian randomization study.

**Methods:**

MR analysis was performed on genome-wide association study (GWAS) summary statistics of GM and EMS. Specifically, the MiBioGen microbiota GWAS (*N* = 18,340) was used as exposure. The FinnGen study GWAS (8,288 EMS cases and 68,969 controls) was used as outcome. We primarily used the inverse variance weighted (IVW) method to analyze the correlation and conducted a sensitivity analysis to verify its reliability.

**Results:**

(1) MR analysis: The results of the IVW method confirmed that a total of 8 GM taxa were related to the risk of EMS. Class-*Melainabacteria* (*p* = 0.036), family-*Ruminococcaceae* (*p* = 0.037), and genus-*Eubacteriumruminantium* (*p* = 0.015) had a protective effect on EMS, whereas order*-Bacillales* (*p* = 0.046), family*-Prevotellaceae* (*p* = 0.027), genus*-Anaerotruncus* (*p* = 0.025), genus*-Olsenella* (*p* = 0.036) and genus-*RuminococcaceaeUCG002* (*p* = 0.035) could increase the risk of EMS. (2) Sensitivity analysis: Cochrane’s Q test (*p* > 0.05), MR-Egger intercept method (*p* > 0.05), and leave-one-out method confirmed the robustness of MR results.

**Conclusion:**

This study performed a MR analysis on two large national databases and identified the association between 8 GM taxa and EMS. These taxa could potentially be utilized for indirectly diagnosing EMS and could lead to novel perspectives in research regarding the pathogenesis, diagnosis, and treatment of EMS.

## 1. Introduction

Endometriosis (EMS) is a common benign gynecological disease, but it has similar manifestations to malignant tumors, such as hyperplasia, metastasis, and recurrence. It affects about 10% of reproductive-age women (Shafrir et al., 2018) and up to 50% of infertile women (Meuleman et al., 2009), which poses a significant global health burden. EMS is defined as the implantation of endometrium-like tissue outside the uterus (usually including the ovary, fallopian tube, and pelvic tissue). Laparoscopy is the preferred diagnostic method, enabling a visual inspection of the affected area (Zondervan et al., 2020). Treatment includes surgical resection of the lesion and hormone drug therapy, but there are side effects and easy recurrence (Chen et al., 2018). There are many theories about the origins of endometriotic tissue. Sampson’s theory of retrograde menstruation is the leading theory. However, it cannot explain why only about 10% of women with retrograde menstruation suffer from EMS (Talwar et al., 2022).

Numerous studies have indicated that an imbalance in the human gut microbiota (GM) could be a significant contributing factor to EMS. GM can affect the growth and diffusion of endometriotic tissue in various ways, as part of the endometriosis micro-environment (Salliss et al., 2021; Li et al., 2022). Leonardi et al. showed that the structure and composition of GM in patients with EMS would change significantly compared with healthy people, EMS is related to the increase of *Proteobacteria, Enterobacteriaceae*, *Streptococcus,* and *Escherichia coli (*Leonardi et al., 2020*)*. Huang et al. (2021) analyzed the composition of microbiota in the gut, cervical mucus, and peritoneal fluid of patients with EMS, and built a classifier model of EMS through a robust machine learning method. They found that GM was more conducive to the diagnosis of EMS than cervical microbiota. Hantschel et al. found that compared with healthy people, GM of patients with EMS was dominated by *Escherichia coli and Shigella* (Hantschel et al., 2019). However, these clinical observational studies cannot clarify the association between GM and EMS, because GM is affected by a variety of factors, including medicine, age, diet, etc.

Mendelian randomization (MR) analysis can reveal the causal relationship between exposure and outcome by using instrumental variables (IVs). Compared with randomized controlled studies, MR analysis can control confounding factors and reduce bias (Emdin et al., 2017). Recent studies have effectively employed MR analysis to examine the association between GM and different diseases (Chen et al., 2022; Liu et al., 2022; Shen et al., 2022). Nonetheless, the relationship between GM and EMS has not yet been investigated. In this study, we selected GM as the exposure and EMS as the outcome for MR analysis to investigate their correlation and provide a theoretical basis for further research on the pathogenesis of EMS. In addition, it can also provide new biological markers, which is helpful in formulating diagnosis and treatment strategies for EMS.

## 2. Methods

### 2.1. Study design and three assumptions of MR

**Figure 1:**
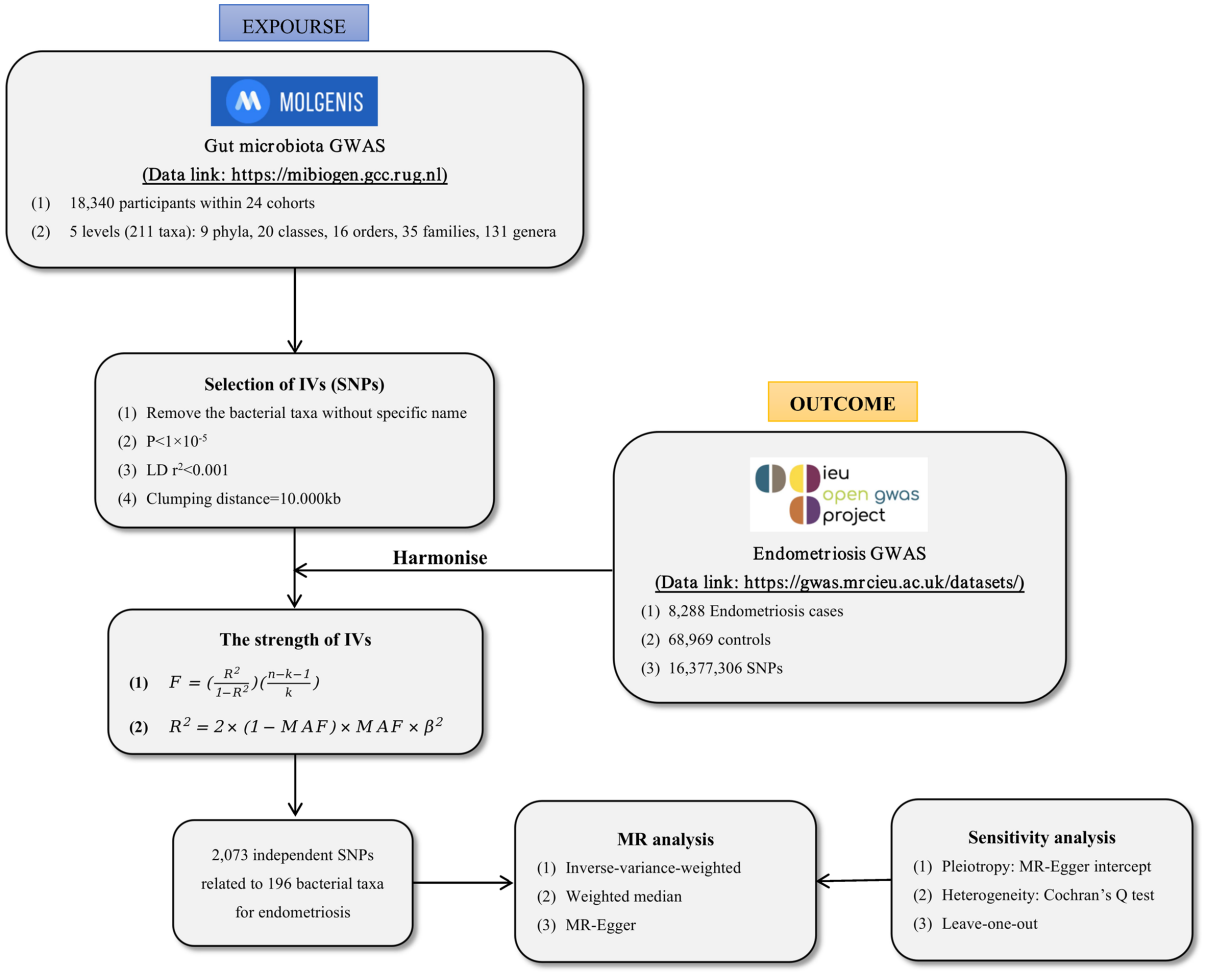
The flowchart of present MR analysis.

We conducted a two-sample MR analysis to investigate the correlation between GM and EMS. The flowchart for the MR analysis is depicted in Figure 1. In addition, to reduce bias and obtain reliable results, we try to satisfy the following three assumptions when using MR analysis: (1) Correlation assumption: IVs are closely related to gut microbiota taxa, (2) Exclusive assumption: IVs do not affect endometriosis through other ways, and (3) Independence assumption: IVs are independent of confounding factors (Davies et al., 2018).

### 2.2. Data source and selection of IVs

#### 2.2.1. Exposure data: GM

We obtained the summary-level data from a GWAS meta-analysis (data link: https://mibiogen.gcc.rug.nl). Kurilshikov et al. (2021) conducted a study on the impact of host genetics on GM composition. They collected genome-wide genotypes and 16S fecal microbiome data from 18,340 individuals across 24 cohorts based on the MiBioGen consortium. A total of 196 taxa have been included in the study, classified under five biological categories: phylum, class, order, family, and genus. It should be noted that participants of European origin were exclusively considered, and 15 GM taxa (unknown family or genus) without specific species names were excluded from the analysis.

To ensure the robustness of data and the accuracy of results, we conducted a quality inspection on the SNPs to obtain qualified IVs. Firstly, we selected IVs at *p* < 1 × 10^−5^ to acquire more comprehensive results (Lv et al., 2021; Liu et al., 2022). Secondly, to reduce the linkage disequilibrium (LD) between SNPs, we performed LD-clumping (*r*^2^ < 0.001, distance = 10,000 kb) on all the IVs, and removed the SNPs that do not conform to the assumption. Thirdly, to prevent alleles from affecting the relationship between GM and EMS, we harmonized the exposure data and outcome data by removing palindrome SNPs.

#### 2.2.2. Outcome data: EMS

We chose the most suitable GWAS from the IEU GWAS database, which we accessed on September 2022 (data link: https://gwas.mrcieu.ac.uk/). We gave priority to GWAS with a large number of samples and European descent queues. Specifically, We used the extract_outcome_Data function in R software to obtain the available complete GWAS summary statistics of EMS in the IEU GWAS database from the FinnGen cohort (GWAS ID: finn-b-N14_ENDOMETRIOSIS) (Garitazelaia et al., 2021). The FinnGen study utilized samples gathered by the National Network of the Finnish Biological Bank. They combined genomic data with EMS, consisting of 8,288 cases and 68,969 controls, and a total of 16,377,306 SNPs. In FinnGen, EMS is classified by using specific codes in the International Classification of Diseases (ICD). In ICD-10, it is defined as N80, in ICD-9 as 617, and in ICD-8 as 6,253 (Li et al., 2023).

### 2.3. Statistical analysis

We applied the “TwoSampleMR” package in the R software (Version 4.2.1) to perform all statistical analyses. We considered a correlation to be statistically significant if *p* < 0.05.

#### 2.3.1. MR analysis

MR analysis was performed after selecting qualified SNPs to determine the correlation between GM and EMS. Since all GM taxa had multiple IVs, we mainly applied the inverse variance weighted (IVW) method to calculate the causal effect value between GM and EMS (Hemani et al., 2018). To determine the magnitude of the effect, the odds ratio (OR) value and 95% confidence interval (CI) are calculated. Additionally, the weighted median (WM) method (Bowden et al., 2016) and the MR-Egger test (Bowden et al., 2015) can be used as supplements to MR analysis. If the number of heterogeneous SNPs exceeds 50%, the WM result is used as a significant causal effect value. If the pleiotropic SNP is higher than 50%, the results of MR-Egger are still valid (Liu et al., 2022).

#### 2.3.2. Sensitivity analysis

For the heterogeneity test, we utilized Cochrane’s Q test. If *p* > 0.05, it is considered that there is no heterogeneity. We also employed the MR-Egger intercept method to test the pleiotropy, and the IVs with *p* < 0.05 were considered to have level pleiotropy. Furthermore, we used the leave-one-out method to detect if there was a significant association influenced by a single SNP. This method helped to further verify the robustness of our data (Xiang et al., 2021).

To test whether there were weak instrumental variables that affected the effect estimates of causality, we used F statistical to test the strength of IVs. F statistical and R^2^ were calculated using the following equations (Palmer et al., 2012; Kamat et al., 2019).


(1) $$F=\left(\frac{R^{2}}{1-R^{2}}\right)\left(\frac{n-k-1}{k}\right)$$

(2) $$R^{2}=2\times \left(1-MAF\right)\times MAF\times \beta^{2}$$

In the above equations, R^2^ represents the variance explained by each IV, *n* represents the sample size, k represents the number of IVs, and the full name of MAF is minor allele frequency.

If the results of the IVW method were statistically significant and there was an absence of heterogeneity and pleiotropy, it is reasonable to infer that GM is linked to EMS.

## 3. Results

### 3.1. Selection of IVs related to GM

After LD-clumping and palindrome removal, we identified 2,075 SNPs as IVs related to 196 taxa for EMS. The F-statistic of rs12938514 was 3.71. This SNP was included in 3 GM taxa (i.e., phylum-*Bacteroidetes*, class-*Bacteroidia,* and order-*Bacteroidales*). After removing it, The final MR analysis included 2,073 SNPs related to 196 GM taxa for EMS. All SNPs showed sufficient validity (F-statistic ranged from 12.18 to 88.43, all *F* > 10), indicating that the effect estimates of causality were unlikely to be affected by weak instrumental variables (Table 1). The key information of IVs is detailed in Supplementary Table S1.

**Table 1:** The number and F-statistic range of SNPs.

Level	Taxa	N.SNP	F- statistic range
Phylum	9	101	16.97–58.16
Class	16	177	13.34–85.38
Order	20	214	16.53–85.37
Family	32	349	12.18–85.37
Genus	119	1,231	14.59–88.43
Total	196	2072	12.18–88.43

### 3.2. Results of MR analysis

**Figure 2:**
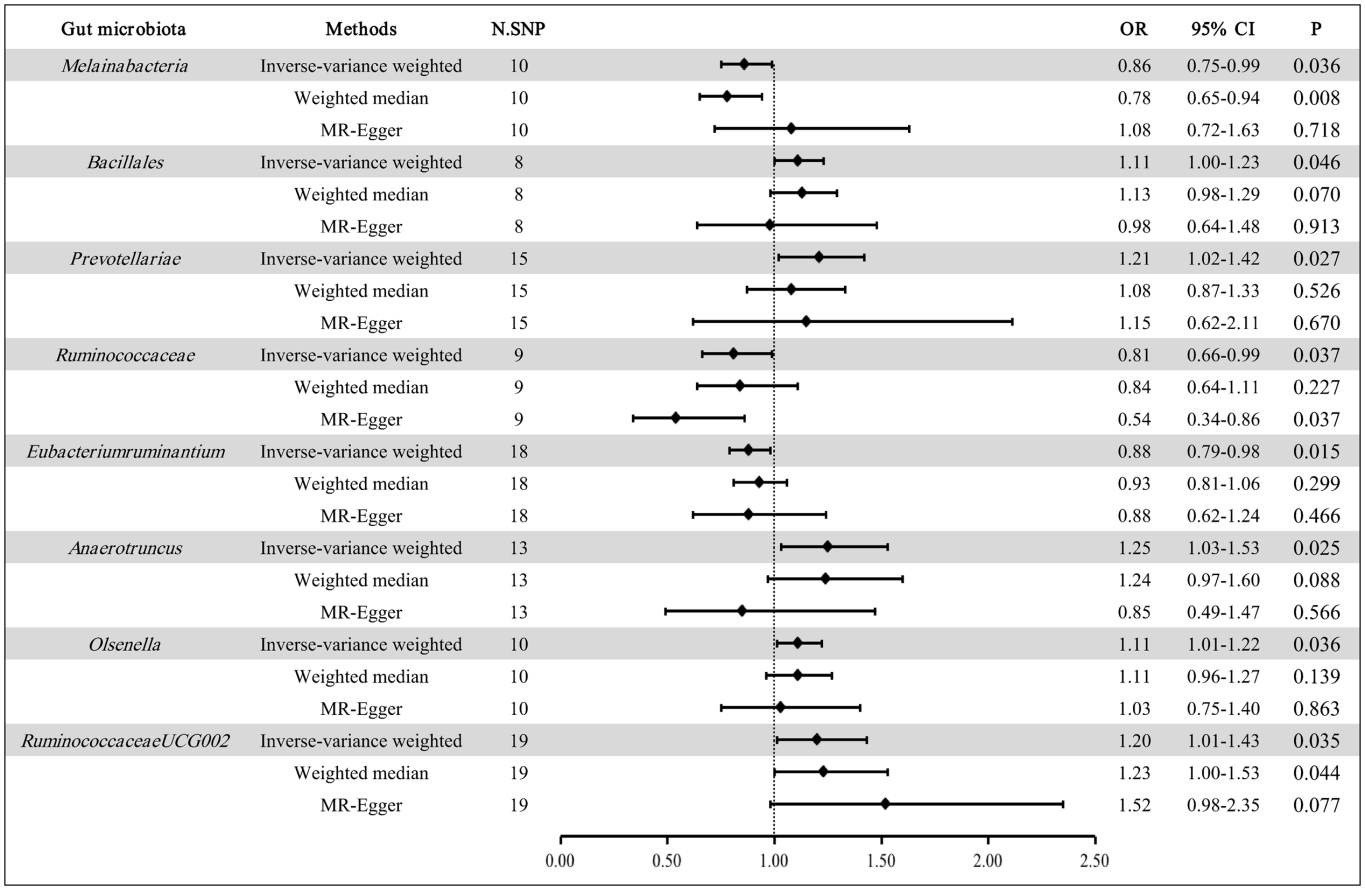
MR results of 8 GM taxa with a causal relationship to EMS.

The results of the correlation between 196 GM taxa and EMS are detailed in Supplementary Table S2. After MR analysis, we determined 8 GM taxa that are relevant to the risk of EMS, composed of 1 class, 1 order, 2 families, and 4 genera (Figure 2). At the biological class classifications level, the results of the IVW method showed that *Melainabacteria* was associated with a lower risk of EMS (OR: 0.86, 95% CI: 0.75–0.99, *p* = 0.036). At the biological order classifications level, we observed that *Bacillales* was associated with a higher risk of EMS in the IVW method (OR: 1.11, 95% CI: 1.00–1.23, *p* = 0.046). At the biological family classifications level, the results of the IVW method indicated that *Prevotellaceae* could increase the risk of EMS (OR: 1.21, 95% CI: 1.02–1.42, *p* = 0.027), while *Ruminococcaceae* could reduce the risk of EMS (OR: 0.81, 95% CI: 0.66–0.99, *p* = 0.037). At the biological genus classifications level, the MR estimates of the IVW method demonstrated that *Eubacteriumruminantium* was negatively correlated with the risk of EMS (OR: 0.88, 95% CI: 0.79–0.98, *p* = 0.015), whereas *Anaerotruncus, Olsenella,* and *RuminococcaceaeUCG002* had positive associations with the risk of EMS (OR: 1.25, 95% CI: 1.03–1.53, *p* = 0.025 for *Anaerotruncus*; OR: 1.11, 95% CI: 1.01–1.22, *p* = 0.036 for *Olsenella*; OR: 1.20, 95% CI: 1.01–1.43, *p* = 0.035 for *RuminococcaceaeUCG002*).

In addition, the results of the WM method supported the relationship between *Melainabacteria, RuminococcaceaeUCG002,* and EMS (OR: 0.78, 95% CI: 0.65–0.94, *p* = 0.008 for *Melainabacteria*; OR: 1.23, 95% CI: 1.00–1.53, *p* = 0.044 for *RuminococcaceaeUCG002*). The relationship between *Ruminococcaceae* and EMS was supported by the MR-Egger test (OR: 0.54, 95% CI: 0.34–0.86, p = 0.037).

### 3.3. Results of sensitivity analysis

Supplementary Table S2 also details the results of pleiotropy and heterogeneity tests for all GM taxa. We tested the reliability of MR analysis results through sensitivity analysis (Table 2). The results of Cochran’s Q test showed that there was no heterogeneity in Melainabacteria (*p* = 0.301), *Bacillales* (*p* = 0.192)*, Prevotellaceae* (*p* = 0.362)*, Ruminococcaceae* (*p* = 0.431)*, Eubacteriumruminantium* (*p* = 0.754)*, Anaerotruncus* (*p* = 0.317)*, Olsenella* (*p* = 0.598), and *RuminococcaceaeUCG002* (*p* = 0.071) for EMS. Meanwhile, the MR-Egger regression method did not provide any evidence of pleiotropy in these GM taxa for EMS (*p* = 0.288 for *Melainabacteria*; *p* = 0.552 for *Bacillales*; *p* = 0.867 for *Prevotellaceae*; *p* = 0.107 for *Ruminococcaceae*; *p* = 0.983 for *Eubacteriumruminantium*; *p* = 0.166 for *Anaerotruncus*; *p* = 0.629 for *Olsenella*; *p* = 0.269 for *RuminococcaceaeUCG002*). In addition, the robustness of the results was further verified by the leave-one-out method (Supplementary Figures S1A–H).

**Table 2:** Sensitivity analysis between GM and EMS.

Gut microbiota	MR-Egger-intercept_P	IVW_Q_P
*Melainabacteria*	0.288	0.301
*Bacillales*	0.552	0.912
*Prevotellariae*	0.867	0.362
*Ruminococcaceae*	0.107	0.431
*Eubacteriumruminantium*	0.983	0.754
*Anaerotruncus*	0.166	0.317
*Olsenella*	0.629	0.598
*RuminococcaceaeUCG002*	0.269	0.071

Therefore, we could believe that *Melainabacteria, Bacillales, Prevotellaceae, Ruminococcaceae*, *Eubacteriumruminantium, Anaerotruncus, Olsenella,* and *RuminococcaceaeUCG002* were related to the risk of EMS.

## 4. Discussion

The study on the correlation between GM and EMS is affected by various confounding factors, so the conclusions of many studies are inconsistent. MR analysis can explore the causal relationship from exposure to results while controlling confounding factors (Emdin et al., 2017). Our study was the first to use a two-sample MR analysis to reveal the association between GM and EMS. By analyzing the summary statistics of GM and EMS GWAS, we identified 8 GM taxa that are associated with the risk of EMS. These findings provide important insights for the prevention and treatment of EMS.

The human gut contains a complex system of microorganisms known as microbiota that play a crucial role in maintaining human health. When there is an imbalance in the microbiota, it can lead to various diseases such as metabolic, immune, and neural-related disorders such as IBD, neurodegenerative diseases, PCOS, and EMS (Strandwitz, 2018; Zhang et al., 2021; Chadchan et al., 2022). More than 90% of GM maintaining the health and balance of the adult intestinal tract are composed of *Firmicutes, Bacteroides, Proteus,* and a small number of *Actinomycetes* (Quaranta et al., 2019). Svensson et al. (2021) used 16S rRNA sequencing to compare the GM between EMS patients and healthy people and found that the ratio of *Firmicutes* to *Bacteroides* in the case group was lower than that in the control group. On the contrary, Shan et al. (2021) found that patients with stage three or stage four EMS had a higher ratio of *Firmicutes* to *Bacteroides* than the healthy control group. In this study, *Bacillales, Ruminococcaceae, Eubacteriumruminantium,* and *RuminococcaceaeUCG002* belong to *Firmicutes*, while *Prevotellaceae* belongs to *Bacteroides.* However, the four GM taxa belonging to *Firmicutes* had opposite impacts on EMS, possibly because GM at the phylum level cover too many taxa, and the interaction between various taxa during the refinement process (e.g., the level of family and genus) may affect the observation of impacts.

In this study, *Ruminococcaceae* showed a negative correlation with the risk of EMS, which is consistent with the results of Huang et al. (2021). Huang et al. (2021) reported that the high abundance of *Ruminococcaceae* was positively correlated with the production of short-chain fatty acids (SCFA) and secondary bile acids (SBAs). In particular, butyric acid, as a SCFA, is believed to counteract gastrointestinal cancer and inflammation (Mao et al., 2021; Wozniak et al., 2022). Therefore, we hypothesize that the decrease of *Ruminococcaceae* may lead to a reduction in the concentration of protective metabolites. This reduction could lead to the development of EMS. However, the effect of butyric acid on female hormone synthesis is bidirectional. In an *in vitro* experiment conducted by Lu et al. (2017), it was discovered that at a lower concentration, butyric acid encouraged porcine granulosa cells (PGC) to secrete progesterone. However, at a higher concentration, butyric acid had a significant inhibitory effect on progesterone secretion. According to research conducted by Liu et al. (2022), it was found that premenopausal women who lack estrogen may develop nonalcoholic fatty liver disease due to the presence of butyric acid. The study also highlighted that butyric acid can regulate estrogen and progesterone levels in females. Interestingly, our study discovered a positive correlation between *RuminococcaceaeUCG002* and the risk of EMS, which contradicts the findings of *Ruminococcaceae*. We suspect that this result may be due to differences in their ability to produce butyric acid, but further investigation is needed to confirm this mechanism.

This study also found that *Eubacteriumruminantium* has the potential to lower the chances of developing endometriosis. This beneficial microbe can reduce intestinal inflammation, as well as improve conditions such as type 2 diabetes and obesity by producing SCFA. Additionally, *Eubacterium* can promote the health of both the intestines and liver by regulating bile acid metabolism (Mukherjee et al., 2020). These findings lead us to believe that *Eubacterium* may reduce the risk of EMS by producing protective metabolites and regulating the host’s metabolism, immunity, and inflammation.

In recent, several studies have indicated that *Bacillus* may pose a risk for patients with encephalitis (Xu et al., 2020) and Graves disease (Yan et al., 2020) when compared to healthy individuals. Additionally, the abundance of *Bacillales* has been found to increase in those with SLE (Xiang et al., 2021), suggesting a potential role in inflammation promotion. These findings align with our research and lead us to believe that *Bacillales* may impact the progression of EMS by promoting pelvic inflammatory damage.

Research has shown that *Prevotellaceae*, a type of bacteria within the *Bacteroides* family, is linked to a higher risk of EMS. This finding aligns with the results of a study conducted by Huang et al. (2021). *Prevotellaceae* is capable of producing an enzyme called β-glucuronidase, which regulates estrogen levels and can convert bound estrogen to free estrogen. The free estrogen is then absorbed into the bloodstream through enterohepatic circulation, resulting in increased estrogen levels and the growth and shedding of ectopic endometrium (Khan et al., 2018). Additionally, *Prevotellaceae* has the potential to release lipopolysaccharide (LPS), which can activate macrophages and trigger the secretion of immune factors, ultimately promoting the proliferation of endometrial stromal cells (Sakamoto et al., 2003).

Recently, scientists have discovered non-photosynthetic *Melainabacteria* in the human gut. This research suggested that *Melainabacteria* could have a significant impact on human health. One advantage of *Melainabacteria* is that it competes for nutrients with cyanobacteria, which produce toxins that can harm the host. This competition can prevent cyanobacteria from occupying a place in the gut, thus protecting the host (Hu and Rzymski, 2022). Our study revealed that *Melainabacteria* is a protective factor against EMS and may be related to its resistance to cyanobacteria. We also found a causal relationship between *Anaerotruncus*, *Olsenella*, and EMS. *Anaerotruncus* belongs to *Verrucomicrobia*, and *Olsenella* belongs to *Actinobacteria*. Both of these bacteria increase the risk of EMS.

In our research, we made several important discoveries. Firstly, we were the first to uncover the link between GM and EMS through a two-sample MR analysis. Secondly, the GWAS database of GM that we used was the most comprehensive in recent years, covering five levels from genus to phylum. This made our study more reliable compared to smaller randomized controlled studies. Lastly, the GM taxa we found to be associated with EMS were completely different from those previously reported, which further emphasized the role of GM in EMS.

Of course, there were some limitations to our study: (1) GWAS in this study only included subjects of European descent, so the results could not be extended to other ethnic groups, (2) We were unable to determine the mutual causality between GM and EMS due to a lack of adequate IVs for reverse MR analysis, and (3) The study did not include analysis at the level of species or strains.

To sum up, we have used two large national databases (MiBioGen database *N* = 18,340 women; FinnGen database *N* = 8,288 women with EMS and 68,969 control women) and discovered a correlation between 8 GM taxa and EMS, which supports other, smaller studies demonstrating a link beween GM and EMS. The taxa we uncovered may become tools for the indirect diagnosis of EMS, while also providing new insights for further research into the pathogenesis, diagnosis, and treatment of the condition.

## Data availability statement

Publicly available datasets were analyzed in this study. This data can be found here: the summary-level data for gut microbiota can be downloaded from the MiBioGen database (data link: https://mibiogen.gcc.rug.nl) and the candidate datasets for endometriosis can be obtained from the IEU GWAS database (data link: https://gwas.mrcieu.ac.uk/).

## Author contributions

Y-LC and XJ designed this study. XJ wrote the manuscript, performed the statistical analysis, and literature search. QY, X-LZ, LX, J-YG, and YR assisted in statistical analysis, reviewed the article and provided critical feedback. Y-LC was responsible for manuscript revision. All authors contributed to the article and approved the submitted version.
