# Supplementary material for: Association between gut microbiota and endometriosis: a two-sample Mendelian randomization study
Source: Front Microbiol. 2023 Sep 27;14:1188458. doi: 10.3389/fmicb.2023.1188458 (PMC10565803; doi:10.3389/fmicb.2023.1188458)
Supplement: Supplementary file 2 [file Data_Sheet_1.docx]

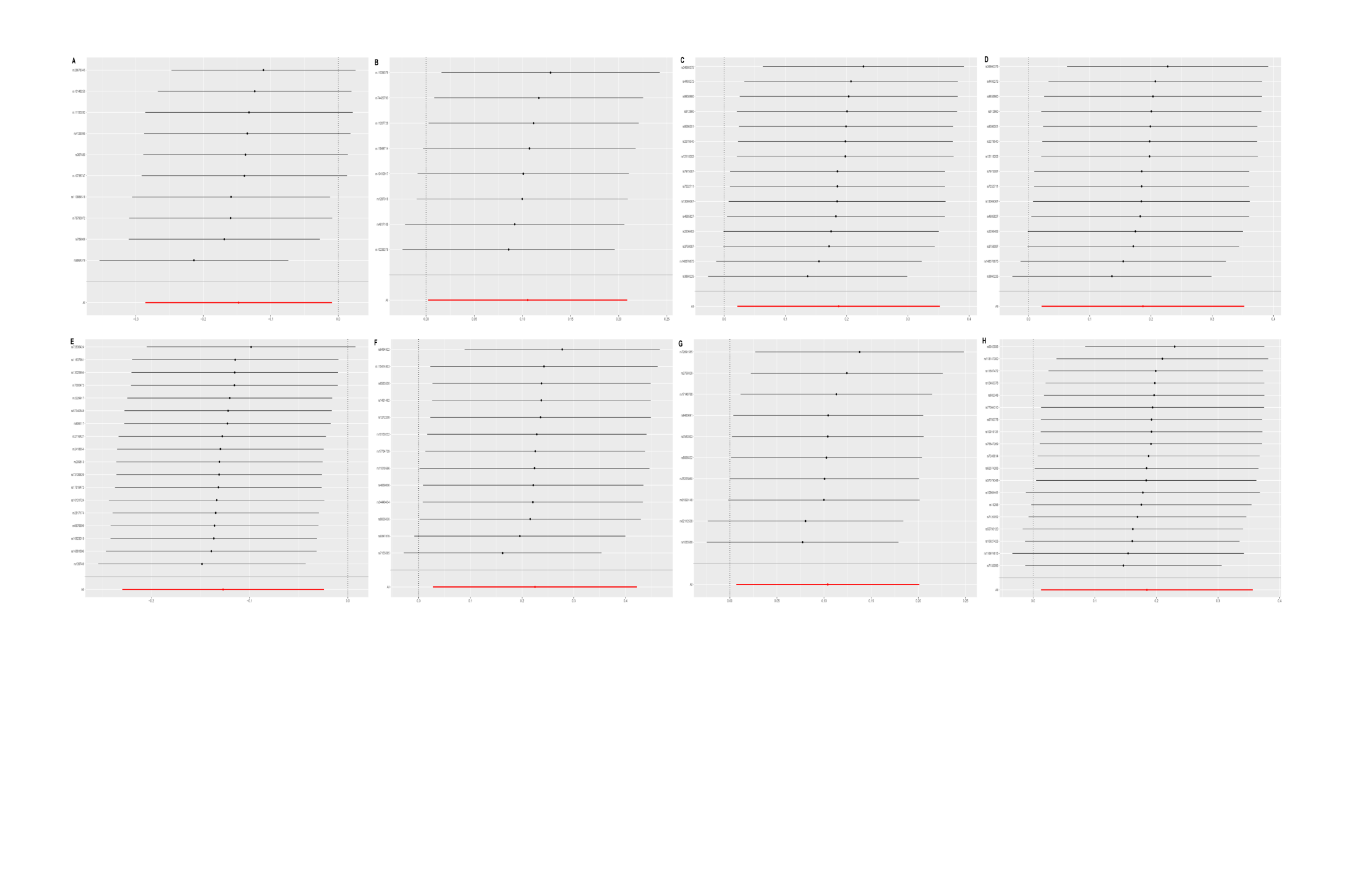


**Supplementary figure 1** The leave-one-out results of GM and EMS. **(A)** The leave-one-out analysis for class-*Melainabacteria* on EMS. **(B)** The leave-one-out analysis for order-*Bacillales* on EMS. **(C)** The leave-one-out analysis for family-*Prevotellaceae* on EMS. **(D)** The leave-one-out analysis for family-*Ruminococcaceae* on EMS. **(E)** The leave-one-out analysis for genus-*Eubacteriumruminantium* on EMS. **(F)** The leave-one-out analysis for genus-*Anaerotruncus* on EMS. **(G)** The leave-one-out analysis for genus-*Olsenella* on EMS. **(H)** The leave-one-out analysis for genus-*RuminococcaceaeUCG002* on EMS.
